# Supplementary material for: Stepwise recombination suppression around the mating-type locus in an ascomycete fungus with self-fertile spores
Source: PLoS Genet. 2023 Feb 10;19(2):e1010347. doi: 10.1371/journal.pgen.1010347 (PMC9949647; doi:10.1371/journal.pgen.1010347)
Supplement: S1 Note — (DOC) [file pgen.1010347.s001.doc]

**1)** We suppose that the number of crossing-overs between the mating-type locus and the centromere is fixed.

Let *n* be the number of crossing-overs between the mating-type locus and the centromere,

and *u* be the frequency of second-division segregation of the mating-type locus.

If the mating-type locus undergoes first-division segregation, an additional crossing-over leads to second-division segregation with probability 1. If the mating-type locus undergoes second-division segregation, an additional crossing-over leads to the maintenance of second-division segregation with probability ½.


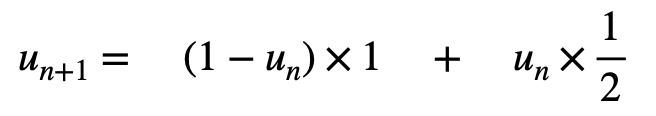


The sequence *u* is arithmetico-geometric, therefore it can be expressed as follows:


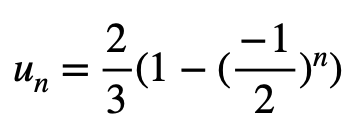


**2)**
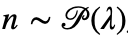
 Now if we suppose that the number of crossing-overs is random and follows a Poisson law of parameter λ:


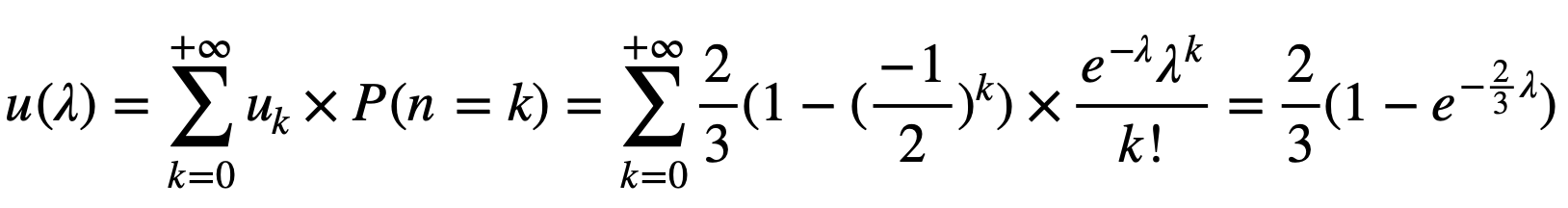
We compute the frequency of

The frequency of second-division segregation *u* is increasing with λ and

**
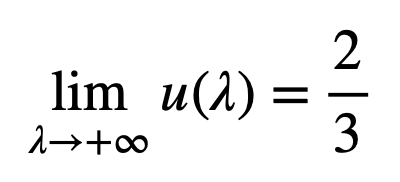
**

Therefore, for any value of λ, *u* ≤ ⅔. Thus, under random segregation of the mating-type locus, the frequency of second-division segregation of the mating-type locus has ⅔ as an upper bound.
