# Supplementary figures and images for: Stepwise recombination suppression around the mating-type locus in an ascomycete fungus with self-fertile spores
Source: PLoS Genet. 2023 Feb 10;19(2):e1010347. doi: 10.1371/journal.pgen.1010347 (PMC9949647; doi:10.1371/journal.pgen.1010347)

Illumina read-based assembly (bp)

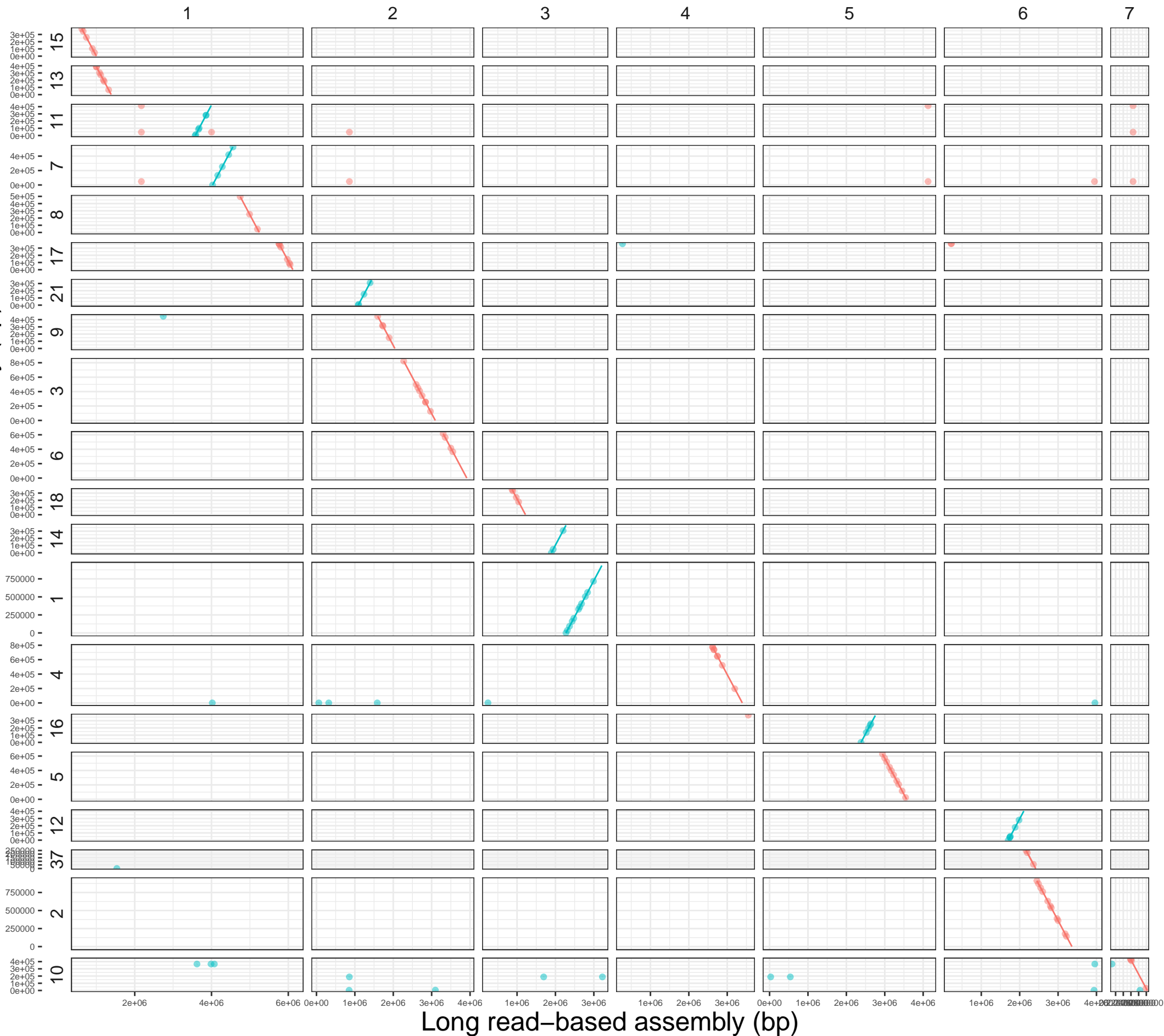

Supplement: S1 Fig — Only the largest contigs (> 400kb) were plotted. Blue and red colours indicate the same and opposite strand collinearity, respectively. Genome collinearity was investigated using the genome sequence aligner nucmer from MUMmer v4.0.0rc1 [2]. (PDF) [file pgen.1010347.s009.pdf]

A

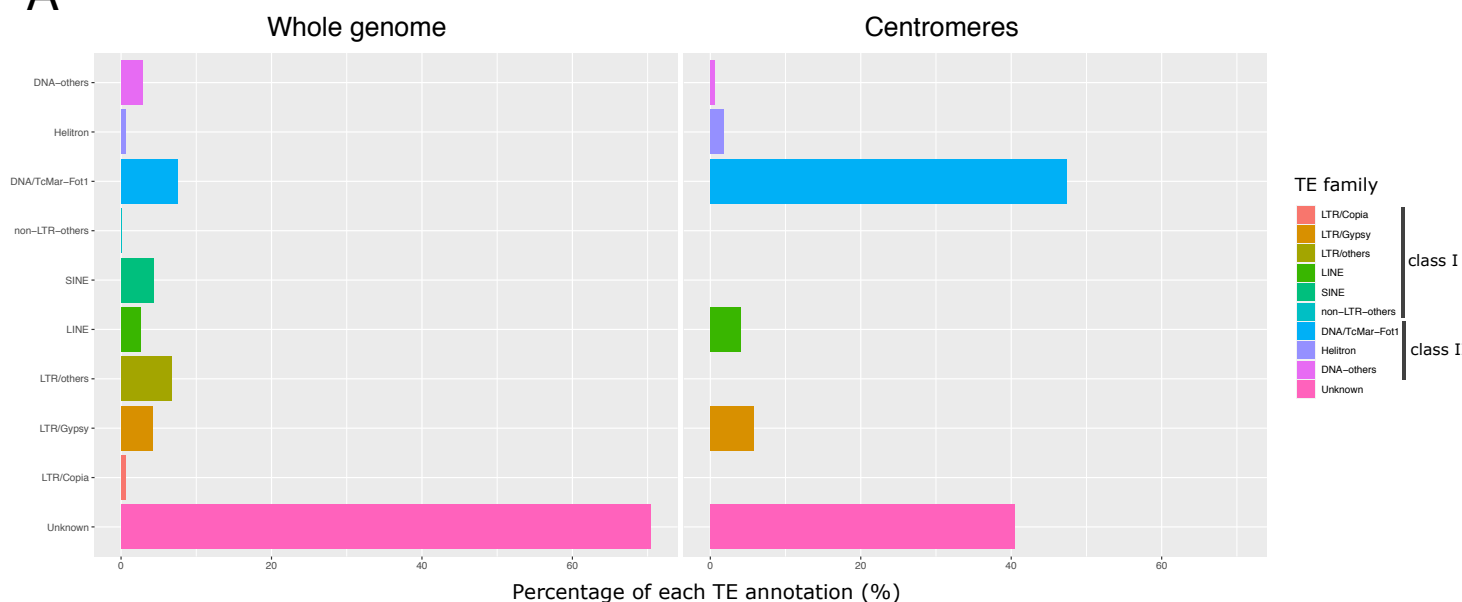

B

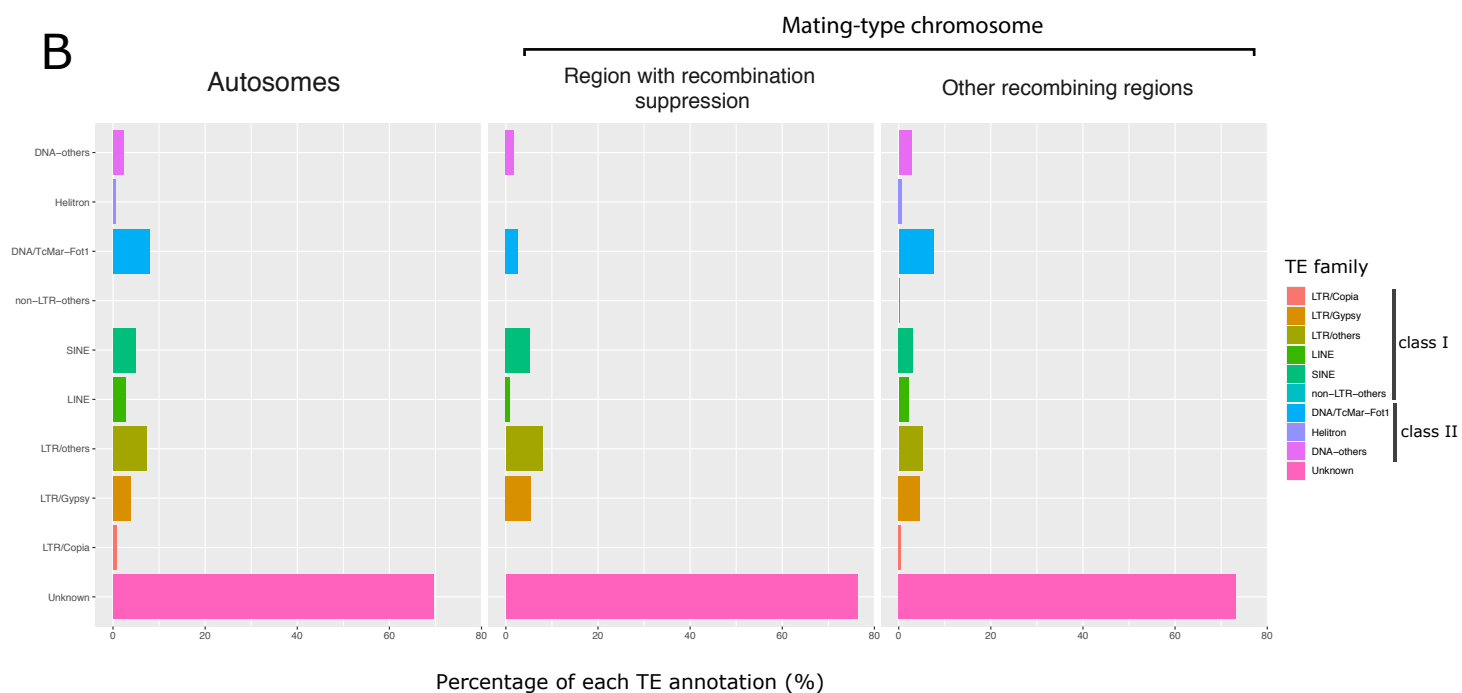

Supplement: S2 Fig — A. Comparison between the whole genome (left) and the centromere (right). B. Comparison between the autosomes (left), the region of recombination suppression around the mating-type locus (middle) and the other recombining genomic regions on the mating-type chromosome (right). (PDF) [file pgen.1010347.s010.pdf]

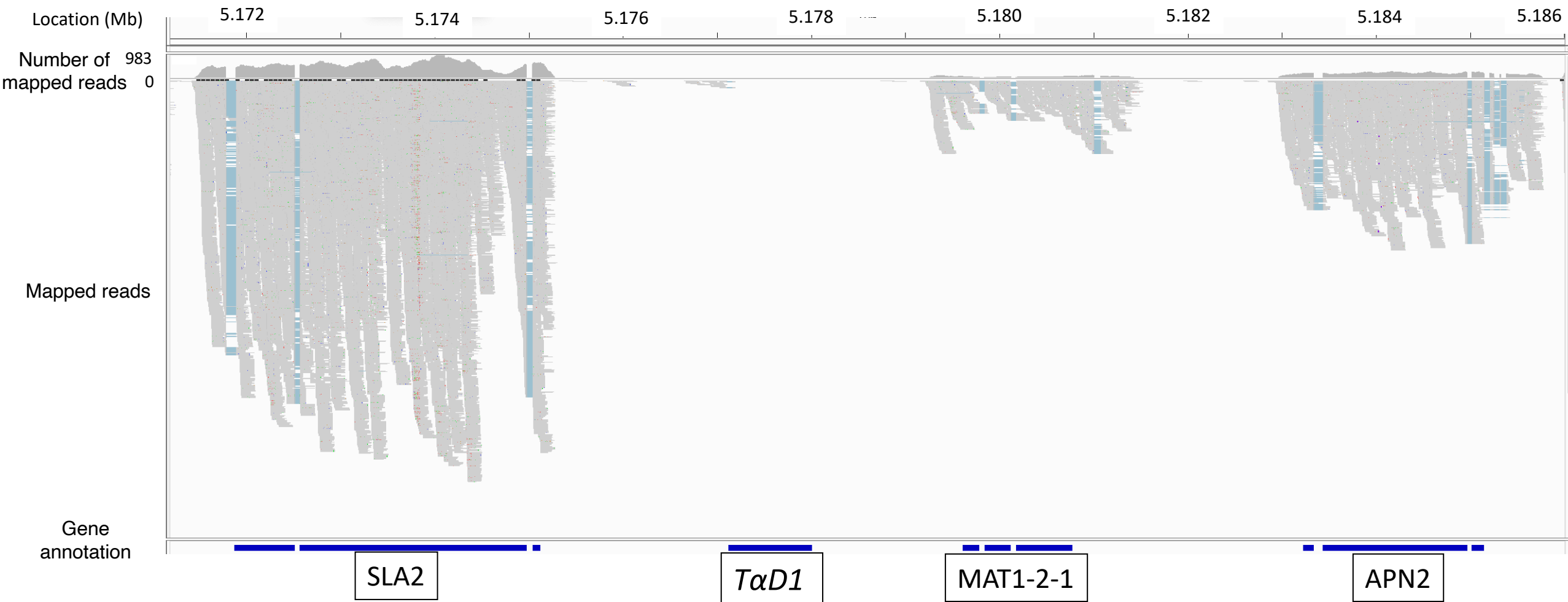

contig 1 of the CBS815.71-sp3 assembly

Supplement: S4 Fig — Visualization of RNAseq read mapping in the Integrative Genome Viewer (IGV) software [5]. The gene names are indicated at the bottom in black rectangles. (PDF) [file pgen.1010347.s012.pdf]

dS value (CBS815.71-sp3 vs. CBS815.71-sp6 genomes)

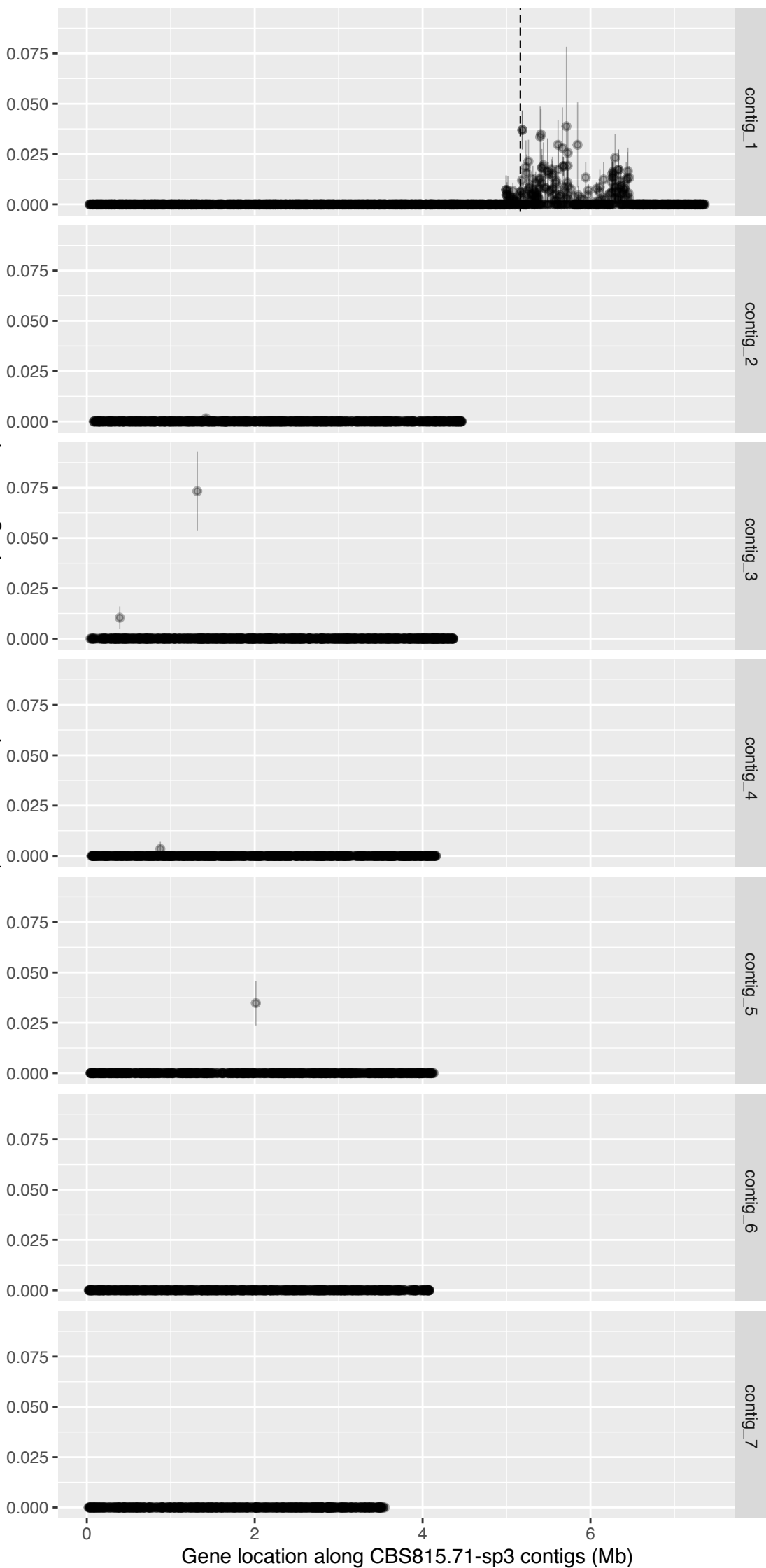

Supplement: S5 Fig — dS was computed per gene between the CBS815.71-sp3 and CBS815.71-sp6 assemblies and plotted according to the CBS815.71-sp3 assembly gene position. Only genes predicted with the same number of exons and the same transcript length were considered for the analysis. The mating-type locus is indicated with a black dotted vertical line and a green triangle. (PDF) [file pgen.1010347.s013.pdf]

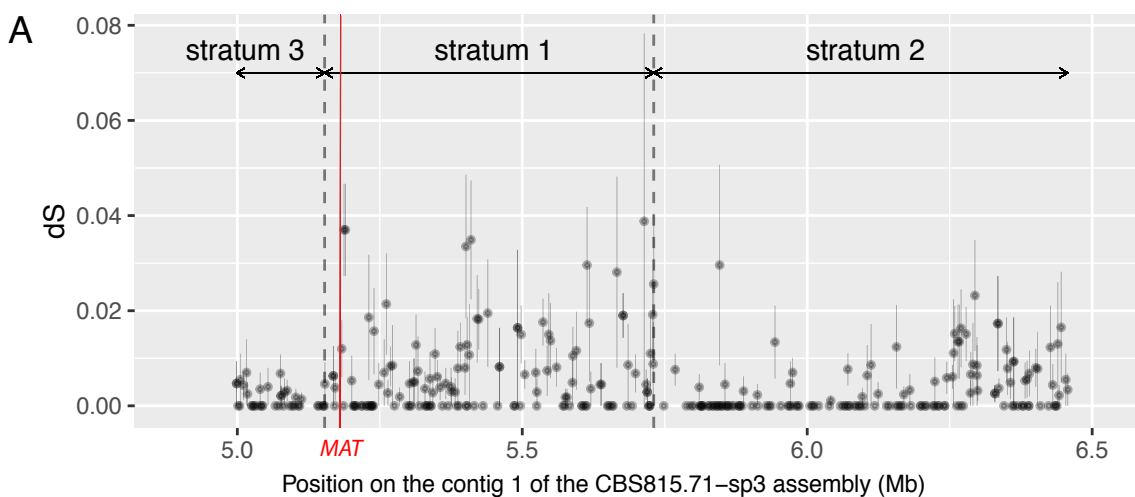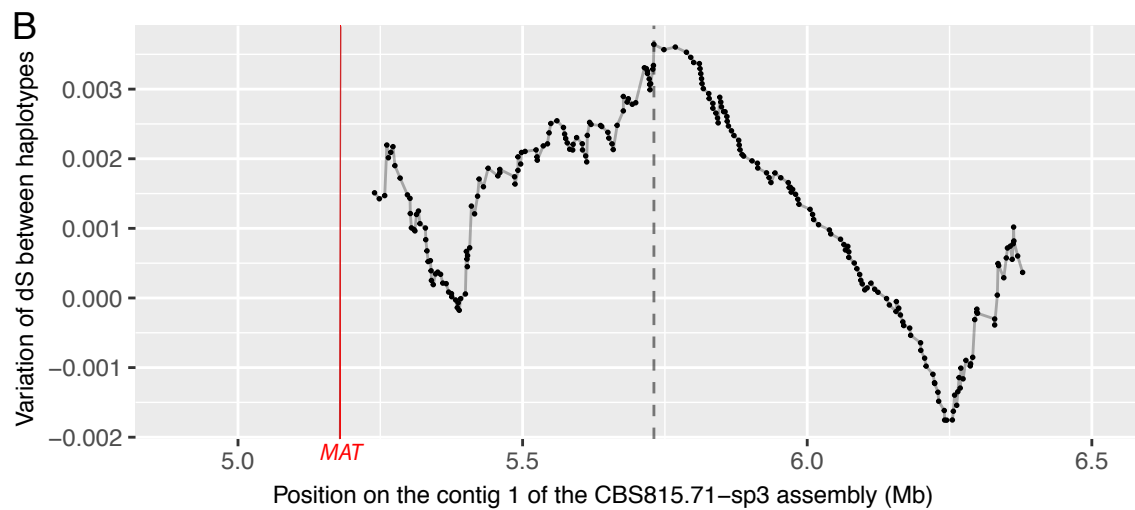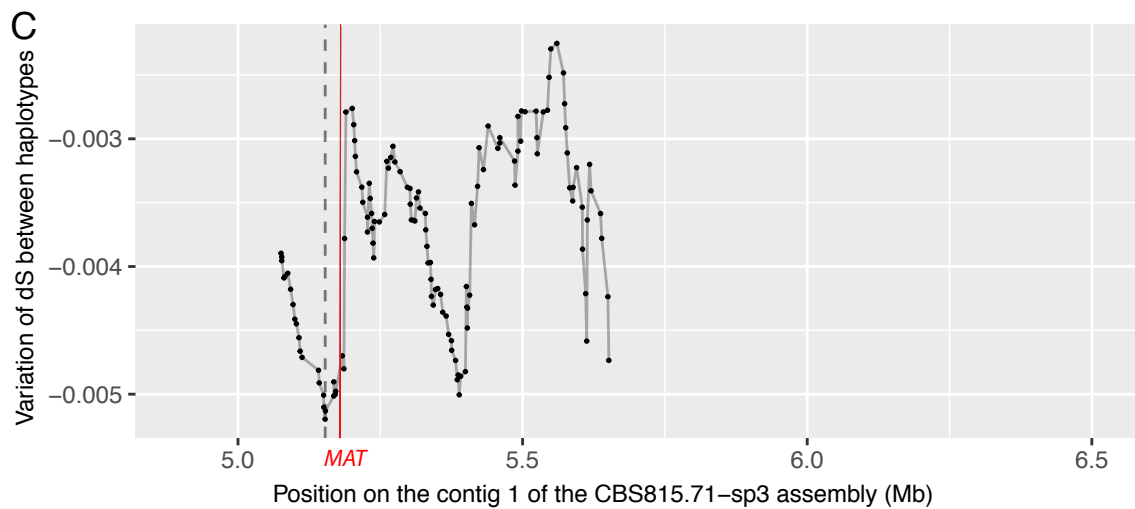

Supplement: S6 Fig — A. Synonymous divergence (dS) along the mating-type chromosome in the Schizothecium tetrasporum CBS815.71 strain and the three identified evolutionary strata 1, 2 and 3 indicated by red, green and blue rectangles respectively. The position of the mating-type locus is indicated by a red vertical line and the positions of the boundaries between strata are represented by dashed lines. The number of genes and average dS within each stratum were computed by excluding the closest gene to each border of the stratum. dS was computed per gene between the orthologs shared between CBS815.71-sp3 and CBS815.71-sp6 assemblies and plotted according to the gene position on the mating-type chromosome of the CBS815.71-sp3 assembly. B-C. Variation of dS in the heterozygous region around the mating type locus when dividing the region into two segments, with the limit sliding along genes, and the difference of dS between the two segments computed for each partition and plotted according to the CBS815.71-sp3 assembly gene position. As described in the main text, two divisions were sequentially performed: (i) between 5,350,000 bp and the end of the heterozygous region to delimit strata 1 and 2; the peak of dS variation at 5,768,335 bp was considered as the boundary between strata 1 and 2 (see panel B); (ii) between the start of the putative non-recombining region and the boundary between strata 1 and 2 to delimit strata 1 and 3; the peak of dS variation at at 5,388,708 bp which was considered as the boundary between strata 1 and 3 (see panel C); Partitions with segments containing less than 10 genes were excluded from the analysis. (PDF) [file pgen.1010347.s014.pdf]

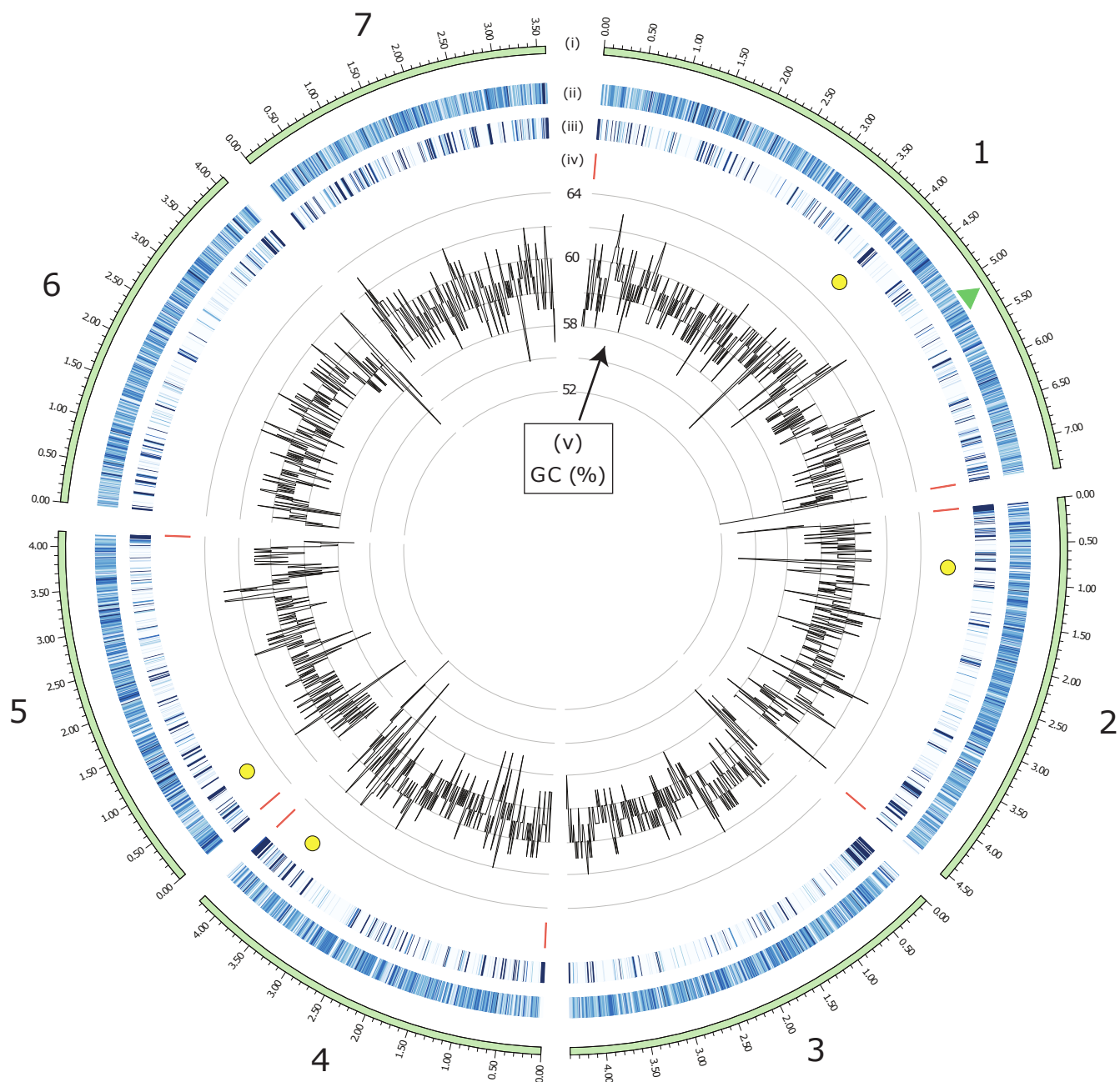

Supplement: S7 Fig — Circular plot of the CBS815.71-sp3 genome showing from top to bottom tracks: (i) Contigs from the CBS815.71-sp3 genome. Only the seven largest contigs were plotted; (ii) Gene density. Mating type locus location was shown with a green triangle; (iii) Transposable element density. Both densities were computed in 10-kb windows overlapping with 1 kb. The colour gradient shows density differences from 0% (light) to 100% (dark). (iv) Location of telomeric repeats (red bars) and putative centromere (yellow circle); (v) GC content (%) computed in 20-kb windows overlapping with 5 kb. (PDF) [file pgen.1010347.s015.pdf]

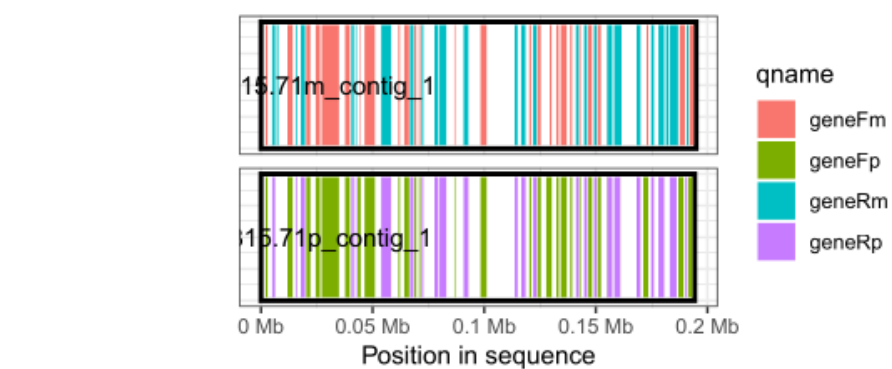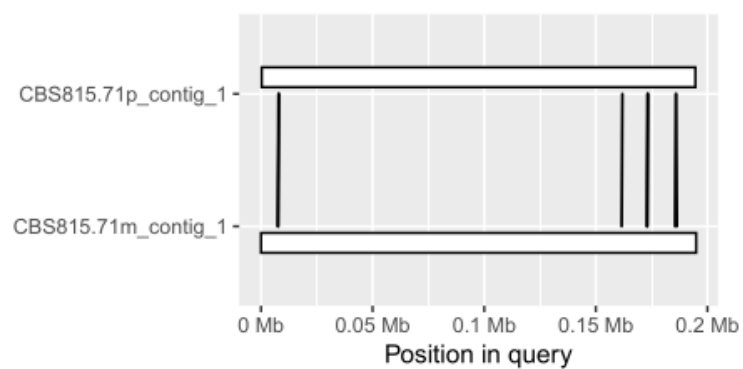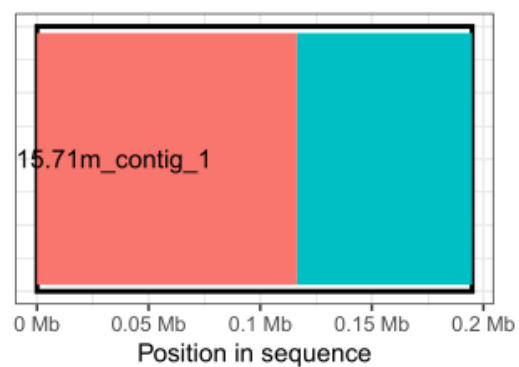

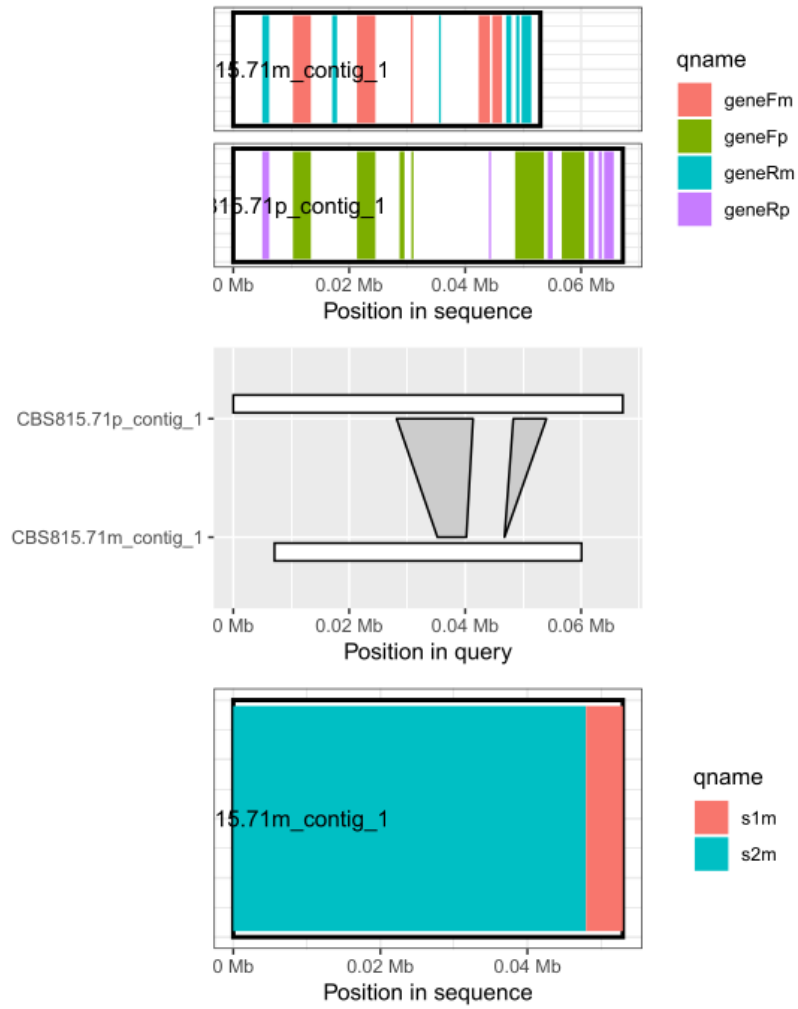

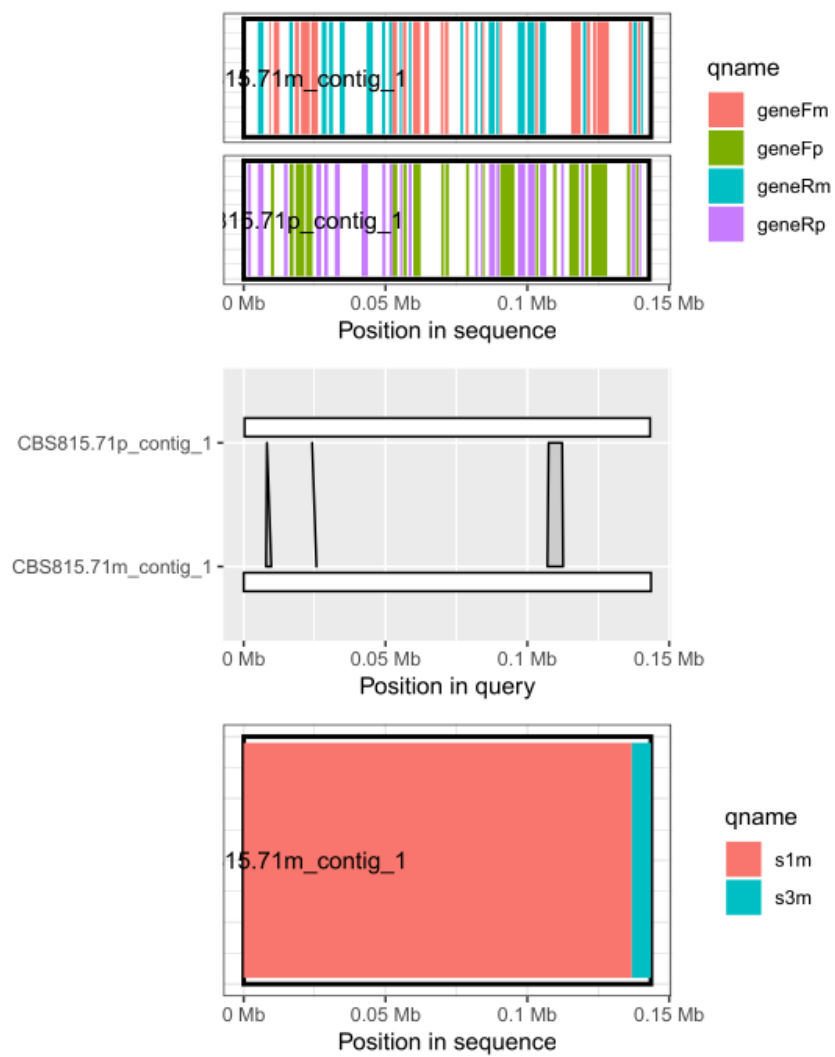

Supplement: S9 Fig — See Fig 5B for a global overview. (PDF) [file pgen.1010347.s017.pdf]
