# Supplementary material for: Stepwise recombination suppression around the mating-type locus in an ascomycete fungus with self-fertile spores
Source: PLoS Genet. 2023 Feb 10;19(2):e1010347. doi: 10.1371/journal.pgen.1010347 (PMC9949647; doi:10.1371/journal.pgen.1010347)
Supplement: S3 Fig — MAT1-1-1 proteins from Schizothecium tetrasporum, Podospora anserina, Neurospora crassa, and TαD1 from S. tetrasporum were aligned with Clustal Omega [3] and coloured with JALVIEW v2 [4]. The colour code of JALVIEW follows the Clustal X default colouring. The 80 residues of the MATα_HMG domain are underlined with a continuous line. This domain is truncated in TαD1. The domain adjacent to the MATα_HMG domain is underlined with a broken line. This domain is present in TαD1. (PDF) [file pgen.1010347.s011.pdf]

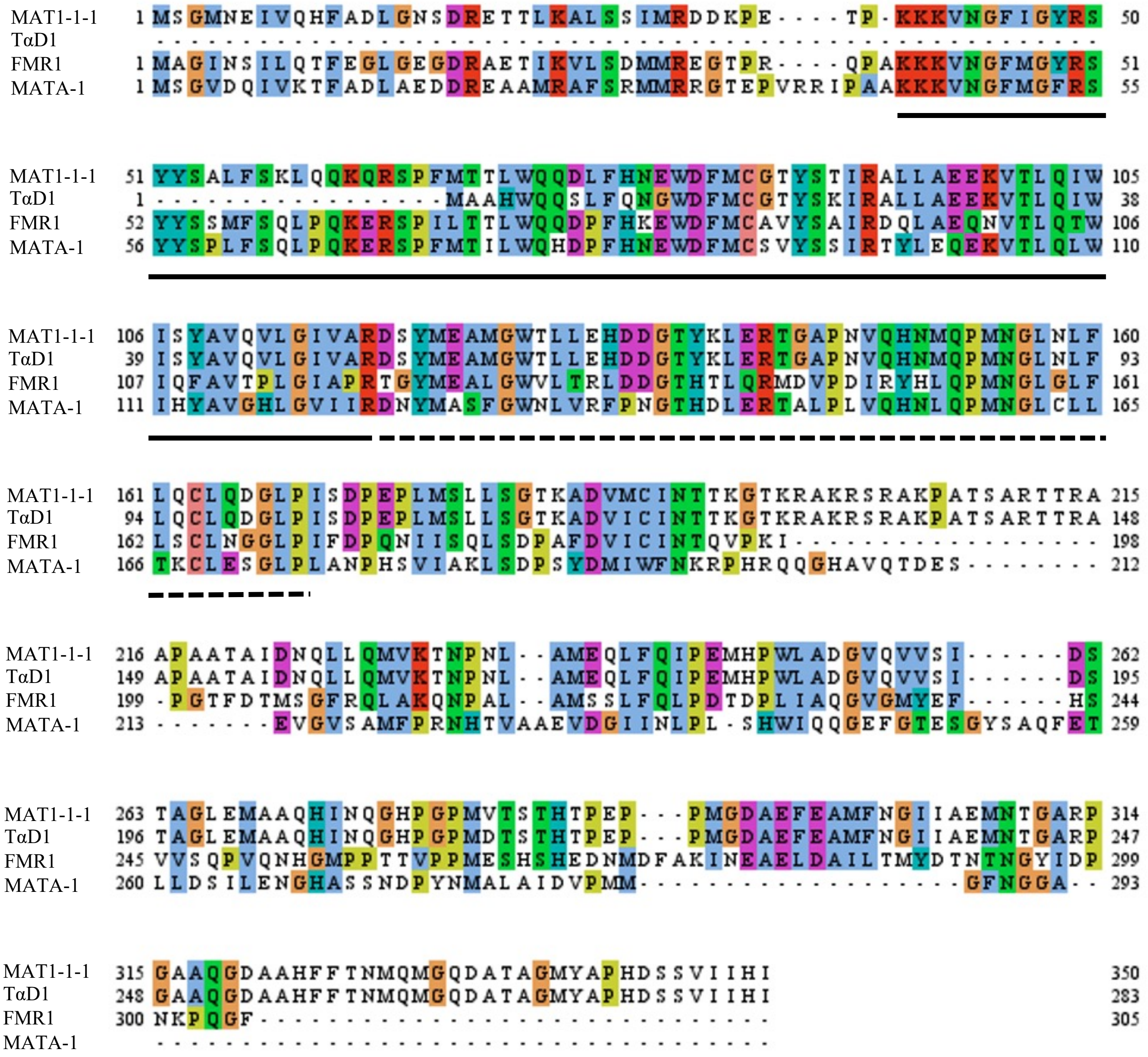

MAT1-1-1 1 MSGMNEIVQHFADLGNSDRETTLKALSSIMRDDKPE----TP-KKKVNGFIGYRS 50
TαD1 ---
FMR1 1 MAGINSILQTFEGLGEGDRAETIKVLSDMMREGTPR----QPAKKKVNGFMGYRS 51
MATA-1 1 MSGVDQIVKTFADLAEDDREAAMRAFSRMMRRGTEPVRRIPAAKKKVNGFMGFRS 55
MAT1-1-1 51 YYSALFSKLQQKQRSPFMTTLWQQDLFHNEWDFMCGTYSTIRALLAEEKVTLQIW 105
TαD1 1 -----------------MAAHWQQSLFQNGWDFMCGTYSKIRALLAEEKVTLQIW 38
FMR1 52 YYSSMFSQLPQKERSPILTTLWQQDPFHKEWDFMCAVYSAIRDQLAEQNVTLQTW 106
MATA-1 56 YYSPLFSQLPQKERSPFMTILWQHDPFHNEWDFMCSVYSSIRTYLEQEKVTLQLW 110
MAT1-1-1 106 ISYAVQVLGIVARDSYMEAMGWTLLEHDDGTYKLERTGAPNVQHNMQPMNGLNLF 160
TαD1 39 ISYAVQVLGIVARDSYMEAMGWTLLEHDDGTYKLERTGAPNVQHNMQPMNGLNLF 93
FMR1 107 IQFAVTPLGIAPRTGYMEALGWVLTRLDDGTHTLQRMDVPDIRYHLQPMNGLGLF 161
MATA-1 111 IHYAVGHLGVIIRDNYMASFGWNLVRFPNGTHDLERTALPLVQHNLQPMNGLCLL 165
MAT1-1-1 161 LQCLQDGLPISDPEPLMSLLSGTKADVMCINTTKGTKRAKRSRAKPATSARTTRA 215
TαD1 94 LQCLQDGLPISDPEPLMSLLSGTKADVICINTTKGTKRAKRSRAKPATSARTTRA 148
FMR1 162 LSCLNGGLPIFDPQNIISQLSDPAFDVICINTQVPKI------------------ 198
MATA-1 166 TKCLESGLPLANPHSVIAKLSDPSYDMIWFNKRPHRQQGHAVQTDES-------- 212
MAT1-1-1 216 APAATAIDNQLLQMVKTNPNL--AMEQLFQIPEMHPWLADGVQVVSI------DS 262
TαD1 149 APAATAIDNQLLQMVKTNPNL--AMEQLFQIPEMHPWLADGVQVVSI------DS 195
FMR1 199 -PGTFDTMSGFRQLAKQNPAL--AMSSLFQLPDTDPLIAQGVGMYEF------HS 244
MATA-1 213 -------EVGVSAMFPRNHTVAAEVDGIINLPL-SHWIQQGEFGTESGYSAQFET 259
MAT1-1-1 263 TAGLEMAAQHINQGHPGPMVTSTHTPEP---PMGDAEFEAMFNGIIAEMNTGARP 314
TαD1 196 TAGLEMAAQHINQGHPGPMDTSTHTPEP---PMGDAEFEAMFNGIIAEMNTGARP 247
FMR1 245 VVSQPVQNHGMPPTTVPPMESHSHEDNMDFAKINEAELDAILTMYDTNTNGYIDP 299
MATA-1 260 LLDSILENGHASSNDPYNMALAIDVPMM-------------------GFNGGA-- 293
MAT1-1-1 315 GAAQGDAAHFFTNMQMGQDATAGMYAPHDSSVIIHI 350
TαD1 248 GAAQGDAAHFFTNMQMGQDATAGMYAPHDSSVIIHI 283
FMR1 300 NKPQGF------------------------------ 305
MATA-1 ------------------------------------
