# Supplementary material for: Stepwise recombination suppression around the mating-type locus in an ascomycete fungus with self-fertile spores
Source: PLoS Genet. 2023 Feb 10;19(2):e1010347. doi: 10.1371/journal.pgen.1010347 (PMC9949647; doi:10.1371/journal.pgen.1010347)
Supplement: S8 Fig — Collinearity between all contigs larger to 500 kb in each assembly. Links showed orthologous genes in the same strand (blue colour) or inverted strands (orange colour) between (i) N. crassa (left) and P. anserina (right); (ii) P. anserina (left) and S. tetrasporum (right); (iii) N. crassa (left) and S. tetrasporum (right). Genome assemblies of the P. anserina S mat+, S. tetrasporum CBS815.71-sp3 and OR74A N. crassa strains were used. (PDF) [file pgen.1010347.s016.pdf]

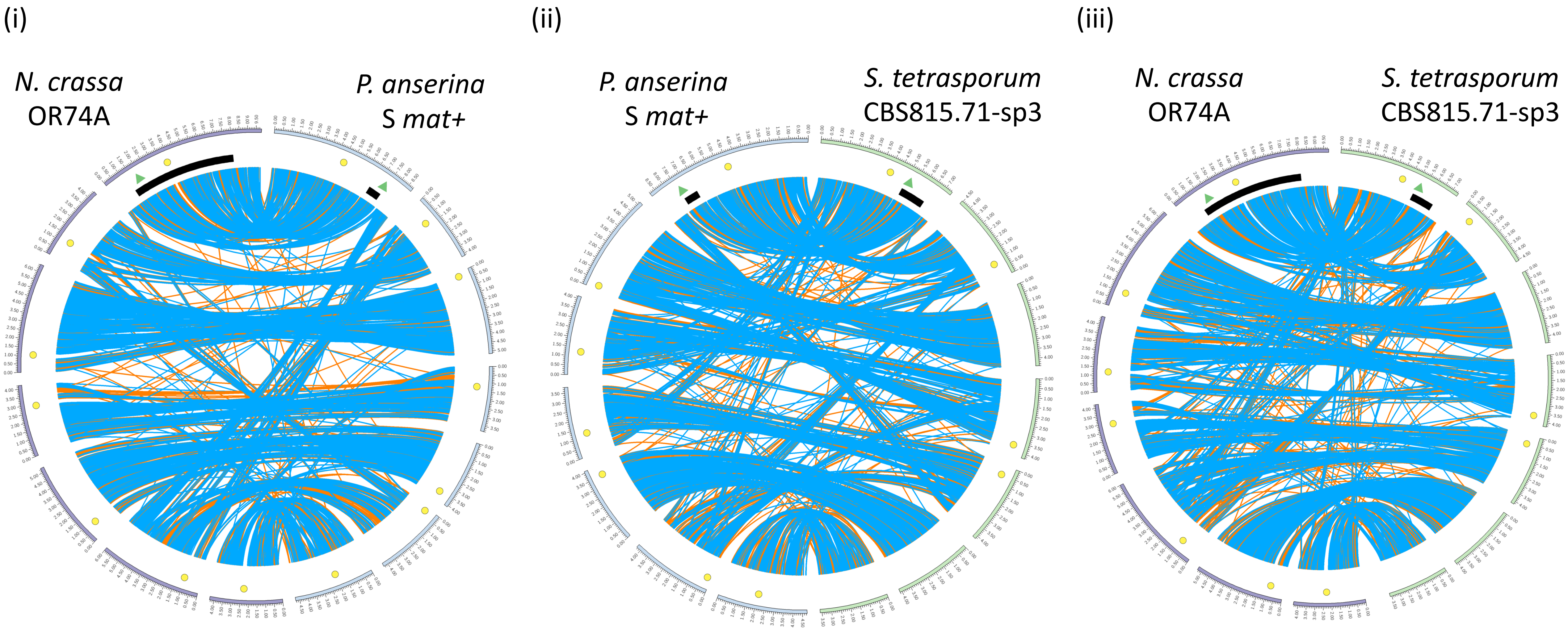
(i)
N. crassa
OR74A
P. anserina
S mat+
(ii)
P. anserina
S mat+
S. tetrasporum
CBS815.71-sp3
(iii)
N. crassa
OR74A
S. tetrasporum
CBS815.71-sp3
