# Supplementary material for: Stepwise recombination suppression around the mating-type locus in an ascomycete fungus with self-fertile spores
Source: PLoS Genet. 2023 Feb 10;19(2):e1010347. doi: 10.1371/journal.pgen.1010347 (PMC9949647; doi:10.1371/journal.pgen.1010347)
Supplement: Attachment — Submitted filename: Answer Letter to Review PLoS Genetics FINAL.doc [file pgen.1010347.s018.doc]

Dear editor,

Thank you very much for taking the time to consider our manuscript for publication in *PLoS Genetics* and for the constructive review process. We have revised the manuscript according to the reviewers’ comments and underlined in yellow the changes in the manuscript. We outline below (in blue) how we addressed all comments. We hope that the revised version will be satisfactory for publication in *PLoS Genetics*.

Note that, in addition to the changes following referees’ comments, we have also improved our gene prediction curation method in the process of addressing a comment by a referee on gene losses in the non-recombining region. We thus had to redo some genomic analyses. This did not change much the general conclusion but actually made the strata delimitation clearer.

We have changed the title and abstract to adapt to the new findings allowed by the analyses on gene losses and this also led to a change in author order.

Best regards,

Fanny Hartmann, on behalf of all co-authors.

Thank you very much for submitting your interesting Research Article entitled 'Stepwise recombination suppression around the mating-type locus in the fungus Schizothecium tetrasporum (Ascomycota, Sordariales)' to PLOS Genetics.

The manuscript was fully evaluated at the editorial level and by three independent peer reviewers who are all experts in the field. The reviewers appreciated the attention to an important problem, and the generation of an important data set for an interesting and unusual pseudo-homothallic fungal species.  They found the paper interesting, and the questions addressed of considerable interest.  They also appreciated the unique and broad data set generated.  This said, they did however raise several substantial concerns about the manuscript as submitted.  Based on the reviews, we will not be able to accept this version of the manuscript, but we would be willing to review a much-revised version.  We cannot, of course, promise publication at that time and a revised manuscript would be subject to review again, ideally with the same reviewers if available.

To summarize comments from reviewers to editor:

The manuscript contains interesting data about the MAT locus of a pseudo-homothallic species, but the interpretations by the authors sometimes seem to be a bit strained or stretched in several areas.  A more realistic interpretation of the data (including instances where the data do not fit a favored hypothesis) would improve this manuscript.

Key and central issues raised by the reviewers include:

1)  What is the nature of the obligate cross over event between MAT and CEN?  Is this at one fixed location and if so, how might it be generated. Alternatively, given the distance between MAT and CEN is this cross over occurring at multiple regions.  The data generated would seem to be sufficient to begin to address this, and consider possible mechanisms.

2) There seems to be some confusion about the description of dS as being high vs low in certain regions, based on comments from two of the three reviewers.

3) The model that strata of recombination is sheltering recessive alleles seems to be more of a model than an interpretation of the data presented.  What is the evidence in support of this model?  Do you observe for example obvious loss of function alleles such as premature stop codons within open reading frames?  One of the reviewers felt that the data presented in fact argued precisely against this model as the findings were not very different in comparing homokaryons and heterokaryons.  This seems a central conclusion of the paper and yet two expert reviewers came to exactly the opposite of the conclusion presented.

4. One reviewer noted:  "if an extended dikaryotic phase facilitates extension or recombination suppression, one might wonder why this is not often the case in basidiomycete mushroom species, many of which spend large parts of their life cycle in the dikaryotic phase."

5.  Another serious concern with data interpretation from one of the reviewers:

"In their analysis of evolutionary strata around the MAT locus, the authors found that there are three strata with the actual MAT locus part of the youngest evolutionary stratum. This is rather surprising and would seem to be a contradiction to the hypothesis that recombination ceased first in the MAT locus region and then suppression of recombination extended outward. The authors argue that a similar situation has been found in Cryphonectria parasitica (lines 582-583). However, in the corresponding paper by Kubisiak and Milgroom, the authors have analyzed C. parasitica strains from different populations and find recombination between the non-recombining region and the MAT locus. In fact, Kubisiak and Milgroom explicitly state that this rejects the hypothesis that this region represents a sex chromosome. Therefore, I don't see the C. parasitica case as confirmation for the authors' hypothesis. This unexpected finding would need another explanation."

**>>We thank the editor for this helpful summary of the main concerns, we explain below the referees’ comments how we addressed these concerns.**

Should you decide to revise the manuscript for further consideration here, your revisions should address the specific points made by each reviewer. We will also require a detailed list of your responses to the review comments and a description of the changes you have made in the manuscript.

If you decide to revise the manuscript for further consideration at PLOS Genetics, please aim to resubmit within the next 60 days, unless it will take extra time to address the concerns of the reviewers, in which case we would appreciate an expected resubmission date by email to plosgenetics@plos.org.

If present, accompanying reviewer attachments are included with this email; please notify the journal office if any appear to be missing. They will also be available for download from the link below. You can use this link to log into the system when you are ready to submit a revised version, having first consulted our [Submission Checklist](http://journals.plos.org/plosgenetics/s/submit-now#loc-submission-checklist).

To enhance the reproducibility of your results, we recommend that you deposit your laboratory protocols in protocols.io, where a protocol can be assigned its own identifier (DOI) such that it can be cited independently in the future. Additionally, PLOS ONE offers an option to publish peer-reviewed clinical study protocols. Read more information on sharing protocols at <https://plos.org/protocols?utm_medium=editorial-email&utm_source=authorletters&utm_campaign=protocols>

Please be aware that our [data availability policy](http://journals.plos.org/plosgenetics/s/data-availability) requires that all numerical data underlying graphs or summary statistics are included with the submission, and you will need to provide this upon resubmission if not already present. In addition, we do not permit the inclusion of phrases such as "data not shown" or "unpublished results" in manuscripts. All points should be backed up by data provided with the submission.

While revising your submission, please upload your figure files to the [Preflight Analysis and Conversion Engine](http://pace.apexcovantage.com/) (PACE) digital diagnostic tool.  PACE helps ensure that figures meet PLOS requirements. To use PACE, you must first register as a user. Then, login and navigate to the UPLOAD tab, where you will find detailed instructions on how to use the tool. If you encounter any issues or have any questions when using PACE, please email us at figures@plos.org.

PLOS has incorporated [Similarity Check](http://www.crossref.org/crosscheck.html), powered by iThenticate, into its journal-wide submission system in order to screen submitted content for originality before publication. Each PLOS journal undertakes screening on a proportion of submitted articles. You will be contacted if needed following the screening process.

To resubmit, use the link below and 'Revise Submission' in the 'Submissions Needing Revision' folder.

********

We are sorry that we cannot be more positive about your manuscript at this stage but do hope that you find the reviews constructive and helpful in revising the manuscript. Please do not hesitate to contact us if you have any concerns or questions.

Yours sincerely,

Joseph Heitman, MD, PhD
Academic Editor
PLOS Genetics

Kirsten Bomblies
Section Editor
PLOS Genetics


Reviewer's Responses to Questions

**Comments to the Authors:
Please note here if the review is uploaded as an attachment.**

Reviewer #1: manuscript PGENETICS-D-22-00843

The manuscript by Vittorelli et al. describes the analysis of the mating type system of the pseudo-homothallic ascomycete Schizothecium tetrasporum. This fungus, which is a member of the Sordariales, produces mostly dikaryotic spores, and the authors showed that the dikaryotic spores carry nuclei with two opposite mating types resulting in a self-fertile dikaryotic mycelium. By isolating the (relatively rare) monokaryotic spores from five-spored asci, the authors were able to obtain homokaryotic strains with different mating types that were used for genome sequencing and analysis of the mating type (MAT) alleles, which are similar to MAT1-1 and MAT 1-2 alleles of other Sordariales. The availability of genome sequences for the mating types allowed the authors to test for recombination suppression in the region of the MAT locus. A 1.3 kb region of suppressed recombination was found extending from the MAT locus towards the telomere.
The results are interesting for researchers working on fungal mating type systems as well as sex chromosomes and related regions of suppressed recombination in general.

**>>We thank the referee very much for the positive comments.**

However, there are several points where the manuscript should be improved:

1. Introduction lines 146-149 and elsewhere: The authors want to test the hypothesis that the long dikaryotic phase in the pseudo-homothallic life cycle might facilitate the extension of recombination suppression around the MAT locus. However, one caveat here would be that to check an actual correlation between pseudo-homothallism and suppression of recombination, one would also need to test more heterothallic species, where one would expect to not find this correlation. As far as I'm aware, this has not been tested in many filamentous ascomycetes, so any correlation with pseudo-homothallism needs to be viewed with caution.

**>>We agree and we have added a sentence at the end of the conclusion (L 751-758). Note however that there are many available genetic maps in heterothallic ascomycetes that do not show any indication of recombination suppression around the mating-type locus; we have also added this point with references L148.**

Also, if an extended dikaryotic phase facilitates extension or recombination suppression, one might wonder why this is not often the case in basidiomycete mushroom species, many of which spend large parts of their life cycle in the dikaryotic phase.

**>>We have tried to clarify this point in the manuscript: as discussed in the Jay et al (2022) paper, many basidiomycetes have multiple alleles at their mating-type loci, in which case we do not expect frequent evolutionary strata. Indeed, even if a fragment with fewer deleterious mutations than average near the mating-type allele can evolve recombination suppression, it will then accumulate further deleterious mutations because of the lack of recombination, until it carries more deleterious mutations than average; it will then be selected against and likely eventually lost, as other mating-type alleles exist, allowing the population to persist without the degenerated allele. The accumulation of deleterious alleles following recombination suppression also occurs in strata near biallelic mating-type loci but neither allele can be lost, as at least two mating-type alleles are required to complete the sexual cycle in heterothallic species; the non-recombining regions therefore remain long enough in the populations to be observed in organisms with only two mating-type alleles. We have added a couple of sentences L163-173.**

2. Results and Discussion: In their analysis of evolutionary strata around the MAT locus, the authors found that there are three strata with the actual MAT locus part of the youngest evolutionary stratum. This is rather surprising and would seem to be a contradiction to the hypothesis that recombination ceased first in the MAT locus region and then suppression of recombination extended outward. The authors argue that a similar situation has been found in Cryphonectria parasitica (lines 582-583). However, in the corresponding paper by Kubisiak and Milgroom, the authors have analyzed C. parasitica strains from different populations and find recombination between the non-recombining region and the MAT locus. In fact, Kubisiak and Milgroom explicitly state that this rejects the hypothesis that this region represents a sex chromosome. Therefore, I don't see the C. parasitica case as confirmation for the authors' hypothesis. This unexpected finding would need another explanation.

**>>We have tried to clarify this point: The model by Jay et al (2022) does predict the occurrence of evolutionary strata that are closely linked, but not completely, to the mating-type locus, as observed here and also in *Cryphonectria parasitica*. Kubisiak and Milgroom in fact found in 2006 a non-recombining region distant by only 3.9 cM from the MAT locus, but** **did not have at the time the Jay et al model to interpret their finding in this context. Provided that the non-recombining fragment is closely linked to the mating-type locus, it can have a sheltering effect (Jay et al 2022). Population genomics data and genome-wide association studies (GWAS) in Stauber et al (2021) (Fig. S1) and Stauber et al (2022) (Fig. S1) show a strong association between the haplotypes of the non-recombining region and the mating-type alleles in *Cryphonectria parasitica*, as the whole non-recombining region was found significantly associated to the mating-type locus in GWAS. This strong association allows deleterious alleles in the non-recombining region to be sheltered by the mating-type alleles, and therefore indicates that a non-recombining region only partially linked to the mating-type locus can evolve via the mechanism modeled in Jay et al (2022). We have added a sentence to clarify this point L672-692. Note however that, with the improved gene prediction curation method, the mating-type locus is just at the limit of the oldest stratum in *S. tetrasporum*, apparently within the oldest stratum. We have therefore changed the discussion accordingly. We still discuss the *C. parasitica* case as the mating-type locus in *S. tetrasporum* is at the limit between the strata 1 and 3.**

3. Lines 305-308 and Figure 3C: In the text, it is stated that CBS815.71-sp6 harbors the MAT1-2 locus, but in the Figure, it's CBS815.71-sp3. Also, the nucleotide positions in Figure 3 are extremely small and difficult to read, it would be better to enlarge these and also to add the contig number for each of the strains.

**>> We thank the referee for having spotted this mistake and for the suggestions. CBS815.71-sp6 harbors the *MAT1-1* allele and CBS815.71-sp3 harbors the *MAT1-2* allele. We corrected the text L344 & L784 and edited Figure 3B and Figure 3C (now Figures 4B & 4C) according to the Referee’s suggestions.**

4. Lines 321-323: It is stated that the transcriptional status of the pseudogene in the MAT1-2 locus is unknown. Could this be checked by using the RNA-seq data that were generated for annotation purposes for the genome sequencing process of this strain?

**>> We thank the referee for this interesting suggestion. We checked read mapping of RNA-seq data generated for annotation purpose against the CBS815.71-sp3 assembly (MAT1-2) and found only few reads mapping at the start of the identified pseudogene sequence TαD1 whereas a substantial number of reads mapped on the mating-type gene *MAT1-2-1* (see the new Figure S4). We added a sentence in the results part (L 358-359) and the material and methods part (L 875-877).**

5. Lines 344-345 and Figure 4: The authors state that lower ds values in the center of the non-recombining region might have occurred through gene conversion. However, in Figure 4 it does not look as if the ds values are lowest in the middle (and consequently higher at the edges), rather they are low in the blue stratum, higher in red and medium in green (with higher towards the edge in the green stratum).

**>> We agree; we meant that dS values appeared, within the green stratum, higher at the inner edge than at the distal edge near the recombining region; however, this was not tested, not very important and was unclear, so we deleted this sentence, and the corresponding sentence in the discussion.**

6. Line 349, Tables S3 and S4: The tables S3 and S4 mentioned in the text were not available in the supplementary material.

**>> We thank the referee for having spotted this mistake. We included now Tables S3 and S4 as separate files.**

7. Introduction lines 119-126: With respect to the two mechanisms for the formation of dikaryotic spores in N. tetrasperma and P. anserina, it might be helpful to have a Figure in the Introduction showing how this works in the two fungi.

**>> This is a nice suggestion, we have added a figure (current Figure 1) and renamed all main figures.**

Reviewer #2: This is a thoughtful research study on a lesser known pseudo-homothallic species of fungus, using a combination of genomics, sequence analysis and experimental evidence to resolve the basis for its reproductive system. Two other species in the Sordariales have been previously studied to account for their pseudo-homothallic life cycles, Podospora anserina and Neurospora tetrasperma. This research explores a third Sordariales species, Schizothecium tetrasporum, and resolves its mating system, to feature a large region of recombination suppression associated with the MAT locus, a key cross over between this region and the centromere, and characterization of the region of suppressed recombination to suggest it arose over three stages of expansion. The research will be of interest to those investigating the basis for mating in fungi, especially convergence in evolution converting presumably heterothallic ancestors into current homothallic species.

There are considerable strengths in the genomics aspect of the research, with high quality genomes (often featuring telomere-to-telomere resolution).

**>> We thank the referee for these positive comments.**

However, the experimental follow up is somewhat limited in its scope. For example, and while the authors acknowledge the limitation, having the set of 190 progeny is a resource that should enable a better understanding of that crossing over event. That is, is this a small ‘hot spot’, or is there sufficient distance between MAT and the centromere for this to occur at multiple sites?

**>> This is indeed an interesting question but we cannot assess this with the material at hand. Indeed, given the homozygosity in the region in the strain analysed here, we cannot use markers in the isolated progeny to perform a more detailed analyses of crossing-over localizations. We have added a sentence in the discussion L647 stating that, in *P. anserina,*  the location of the crossing-over is not a single, localized hotspot of recombination (Marcou et al. 1979; Espagne et al. 2011 ; Hartmann et al 2021).**

Second, the loss of nuclei and nature of the hyphal stages is of interest in terms of the level of stability of the homothallic state, potentially resolving the authors’ question of the maintenance of a dikaryon (e.g., do the cells really contain two nuclei as illustrated in figure 2?). Here it may have been worth exploring with microscopy on cultures looking at nucleus numbers vs. septation.

**>> We thank the referee for the suggestion and we have tried some experiments. However, understanding the sorting of mat1-1 vs mat1-2 nuclei as homokaryons requires a dynamic analysis of their movements in apical hyphae, which is beyond the scope of this paper, as it will need the setup of a transformation system that will enable the labelling of nuclei with different fluorescent makers (e.g., GFP and mcherry). This has been added to the manuscript L286-288.**

One last point is it is curious how different people interpret the same data differently. The results discussed in lines 514-550 of this study, to this reviewer at least, are very strong evidence counter to the hypothesis of sheltering deleterious alleles, since by and large there is not a huge different between the homokaryons and the heterokaryon.

**>> We have tried to be clearer on this point: L598-609; L680-692. We do not necessarily expect a huge different between the homokaryons and the heterokaryon of one strain under the deleterious sheltering hypothesis in our experimental setting because: i) the difference in fitness in the model is an average across all strains in the populations, it may not apply to one particular strain; ii) fitness differences can be very small for the process to work; iii) it is unlikely that our experiment has captured all conditions or stages in which the fitness differences may occur. We have also added a sentence in the conclusion explicitely stating that there is no definitive evidence here indeed in favor of the deleterious mutation sheltering hypothesis, but that the association between pseudo-homothallism (or an extended dikaryotic phase associated with a biallelic locus) and recombination suppression is a prediction of the model; L751-758. Note however that the analyses that have been added following referees’ comments revealed gene losses in the non-recombining region, and precisely at the evolutionary strata margin.**

The third hypothesis (lines 536 onwards) is intriguing, but has the same challenge that it is difficult to see a difference between homo- and heterokaryons.

**>> We agree and this is stated in the paragraph: « However, there is no evidence of antagonistic selection between mating types for these functions and we found no phenotypic differences between homokaryons of opposite mating types. »**

Alternative explanations exist, from the perhaps unpopular concept of ‘neutral theory’ through to the potential selective advantage the ascospores cell type itself might play in the lifecycle.

**>> We fully agree. The first corresponds to the hypothesis of the selective advantage of a self-fertile spore, if we understand correctly the Referee’s comment, and this is discussed in the manuscript, for example L556-557 : « The resulting self-fertile heterokaryotic ascospores allow reproductive insurance in short-lived habitats such as dung» , L680-681 : « The selective advantage of producing self-fertile spores would then be the driver to develop this mechanism « and L695-696 : « The selective pressure for this stepwise increase would be to reach a higher number of dikaryotic ascospores growing into self-fertile mycelia ». Regarding the neutral explanation, we have added sentences and references L616-619 and L697.**

The data suggest that Schizothecium tetrasporum is facultatively pseudo-homothallic/heterothallic.

**>> This is indeed interesting to explicitely state, and we have added this term L724.**

A few minor grammar/text considerations are as follow.

Line 36: ‘convergent association’ is unclear in meaning.

**>> « convergent » was indeed redondant and has been deleted**

Line 50: too many adjectives, probably ‘sheltering deleterious alleles’ is better.

**>> This has been corrected as suggested.**

Line 54: ‘mutations’ plural.

**>> The typo has been corrected**

Line 86: rewrite to remove double ‘associative’ ‘associated’.

**>> We have reformulated the sentence**

Line 151: remove italics from ‘var.’,

**>> The typo has been corrected**

Lines 161-163: choice of words suggests reference 21 was not inadequate, so recommend deleting ‘However’ and ‘clearly’.

**>> Done**

Line 183: delete ‘also known as P. tetraspora’.

**>> Done**

Line 272: delete ‘to our knowledge’.

**>> Done**

Line 312: remove italics from ( ).

**>> Done**

Line 342: add space ‘and 6.457’.

**>> Done**

Line 378: remove italics from ‘spp.’.

**>> Done**

Line 420: for consistency in style with the rest of the text, ‘Sordariales’ should be in italics in the subtitle.  **>> Done**

Line 468: perhaps use a more descriptive subtitle.

**>> We have changed the subtitle to: « Striking evolutionary convergence of breeding systems and recombination suppression across distant fungal lineages »**

Lines 553-562: three uses of ‘proximal’, probably all can be deleted?

**>> We think that it is important to distinguish the proximal and evolutionary causes for phenomena, especially as we discuss the two types of explanations in the manuscript, otherwise it can lead to misunderstanding. We have replaced one « proximal » term by « molecular » to avoid too many repeats.**

Lines 563-565: worth re-writing, as duplicated ‘convergent evolution’.

**>> Indeed, we have replaced one by « occurrence »**

Line 858: add initials to author.

**>> done.**

Line 893: capital ‘P’ in two places.

**>> done**

Figure 2B: ‘apoptosis’ refers to the whole cell, rather than an organelle.

**>> We replaced the term ‘apoptosis’ by ‘loss of nuclei’.**

Reviewer #3: This is an intriguing manuscript. The authors investigated the breeding system of the filamentous fungus Schizothecium tetrasporum for the first time. They chose this fungus because it enables them to test their hypothesis that there is an evolutionary association (by convergent evolution) between pseudohomothallism (which involves dikaryotic nuclei of opposite MAT genotypes) and the existence of a large block of suppressed recombination around the MAT locus. The authors’ group previously proposed this hypothesis in ref 14 based on observations from two pseudohomothallic species, Podospora anserina and Neurospora tetrasperma. Now, in this study they investigated a third pseudohomothallic species, Schizothecium tetrasporum, and find that it too has a large non-recombining region around the MAT locus. Importantly, these three fungi are not closely related to each other (they are in different families).

The results are certainly interesting. The authors have done a lot of work to establish a new genetic system for studying this species: as initial work for this manuscript, they found conditions for growing and mating it, they identified a pair of parental strains that can be crossed, and they sequenced the genomes of the parental strains. The work is scientifically rigorous. The authors show suppression of recombination both in a laboratory cross (page 15) and by evolutionary analysis of synonymous sequence divergence (Fig 4), and there is good congruence between the size of the non-recombining region from these two methods. The manuscript is very well written (the Abstract and Introduction were exceptionally clear – thank you!).

**>> Many thanks for this very positive assessment!**

The most intriguing aspect of this manuscript for me was the occurrence of a “single and systematic” crossing-over event between the centromere and the MAT locus, in both Schizothecium tetrasporum and Podospora anserina, leading to second division segregation (SDS) of the MAT locus in these species (as opposed to first division segregation in Neurospora tetrasperma). I am left wondering about the molecular mechanism of this single crossover, which occurs in more than 99% of meioses (Line 222). The authors do discuss this issue (Lines 493-509), but it wasn’t clear to me if nothing is known about the molecular mechanism in P. anserina (where the SDS was discovered years ago, I think).

**>> The high SDS of the mating type was indeed discovered in *P. anserina* years ago. Despite several experimental tests, the mechanism is still unknown, as now indicated lines 647. We have added some additional information in the lines 574-580 to avoid confusion with the concept of “obligate CO”, which has a different, specific meaning in the field of meiosis.**

Line 344 and Figure 4. I’m confused by the statement “the dS values were lowest in the center of the non-recombining region”. Looking at Figure 4, the opposite seems to be true because the highest dS values are in the red region (stratum 1) which is surely the center of the non-recombining region. In fact, Figure S6A shows that the median dS in this region is the highest. However, I don’t think that “lowest” on line 344 is a typo, because it is repeated on line 596/597. So I don’t understand what the authors are talking about; maybe an arrow pointing to the “low” region in Figure 4 would help.

**>> As answered above to the Referee 1, we meant that dS values appeared, within the green stratum, higher at the edge than at the inner side; however this is not very important, not tested and seemed unclear, so we deleted this sentence, and the corresponding sentence in the discussion.**

Lines 328-400 (dS analysis section). The authors like the hypothesis that evolution selects for recombination suppression as a way of sheltering recessive deleterious alleles by linking them to a permanently heterozygous locus (MAT) (Lines 137-140, and 514-526). If this hypothesis is true, wouldn’t you predict that some of the 400 genes in the non-recombining region might contain obviously deleterious mutations (e.g. internal stop codons) in one allele, to a greater extent than in the rest of the genome? Did you look for such a pattern, and if so what did you find?

**>> Thanks for the very interesting suggestion. We performed in-depth analyses and actually found signs of degeneration in terms of gene losses and gene disruption; we have added this information in the paper and reformulated some of the discussion accordingly. See L441-454, L598-604, and Figure 5A.**

Lines 410-418. The text here is difficult to understand without looking at Figure S7, because the locations of the P_NRR markers are not shown in Figure 4. Please consider merging these two figures. Remember that many readers will not look at supplementary figures.

**>> We thank the reviewer for the suggestion and merged Figure 4 and Figure S7 as a single figure (currently Figure 5) to improve the clarity of the text for readers.**

Lines 420-464. I didn’t really understand the purpose of this whole section about synteny of genes in the non-recombining regions (NRR) of the different species. Are you testing an hypothesis here? Some introductory sentences might help. After reading the Discussion, I think maybe that this section is connected to the “supergenes” idea on Lines 538-543, but it’s not clear.

**>> That’s right, we have clarified this point L476-481, but also that the supergene hypothesis is more about shared gene content in the non-recombining region than synteny.**

Did you test whether interspecies synteny is more conserved in the NRR than elsewhere in the genome?

**>> This is a good point and we have added sentences in the results (L513) and the discussion L632-637. In addition, we have added a sentence to highlight that synteny is also conserved in non-pseudo-homothallic species L522. We have also clarified that the supergene hypothesis is more about shared gene content in the non-recombining region than synteny, and that it actually appears not particularly supported in our analyses L476-481; L517-523.**

Lines 507-514 are slightly unclear. Line 514 begins “An alternative hypothesis...” but it wasn’t obvious what the first hypothesis was. Reading back over lines 507-510, I wonder if the authors’ intention would be clearer if the sentence beginning on line 510 was changed to “One hypothesis could therefore be that suppression of recombination around the mating-type locus may be directly linked to the mechanism that prevents a second CO...”.

**>> Changed as suggested**

==

Graphical summary: I suggest changing “autofertile” to “self-fertile” because that is the phrase used in the text.

**>> Done**

Lines 104-126. Does all the information in this paragraph come from reference 21, or should some other papers be cited?

**>> We have added references as suggested.**


Lines 121 and 125. I wasn’t sure if there is a difference between “parallel” spindles and “aligned” spindles. If they’re synonyms, please use the same word in both places. If there is a difference, please explain it and why it is important. (Also occurs on line 161).

**>> We have tried to clarify the difference L128 and L133 and added a new figure to illustrate the text (Figure 1).**


Lines 135-137. Are non-recombining regions around the MAT locus absent in heterothallic species?

**>> We have added a sentence to clarify this point :** **«** **There is indeed no recombination suppression around the mating type reported so far in true heterothallic ascomycetes as seen in available genetic maps (29–35); an exception is *Cryphonectria parasitica* (36), in which dikaryons are nevertheless relatively frequently isolated in nature (37–39)”, see  L146-149**

Lines 150-158. The three species are in different families. I presume that they each evolved pseudohomothallism separately, but I did not see a statement in the manuscript saying that this is the case (or that the authors assume it to be the case).

**>> This is indeed an important point, we have added a sentence to clarify this: «The substantial phylogenetic distance between these pseudo-homothallic species, that are each closely related to true heterothallic and homothallic species, indicate that this breeding system has evolved independently in these three lineages.”**  **L186-189.**

Line 307. Is there an error on this line? It says that CBS815.71-sp6 is MAT1-2, but Line 201 and Figure 3 say that it is MAT1-1.

**>> We thank the reviewer for pointing out this mistake and we corrected the text.**

Figure 4, please label the strata 1, 2 and 3.

**>>We labelled the strata and added an inset showing a zoom in the non-recombining region, representing the mean dS per stratum with horizontal lines as (see the current Figure 5A).**


Typos:
Line 54, should be “mutationS”

**>> Corrected**

Line 512, should be FDS

**>> Corrected**
