# Supplementary material for: Stepwise recombination suppression around the mating-type locus in an ascomycete fungus with self-fertile spores
Source: PLoS Genet. 2023 Feb 10;19(2):e1010347. doi: 10.1371/journal.pgen.1010347 (PMC9949647; doi:10.1371/journal.pgen.1010347)
Supplement: Attachment — Submitted filename: Answer 2nd revision-v3.doc [file pgen.1010347.s019.doc]

Dear editor,

Thank you very much for the time spent on our manuscript for publication in *PLoS Genetics* and for the fast and constructive review process. We have revised the manuscript according to the reviewers’ comments and underlined in yellow the changes in the manuscript. We outline below (in blue) how we addressed all comments and we have uploaded a “striking image”. We hope that the revised version will be satisfactory for publication in *PLoS Genetics*.

Best regards,

Fanny Hartmann, on behalf of all co-authors.

**Thank you very much for submitting your revised Research Article entitled 'Stepwise recombination suppression around the mating-type locus in an ascomycete fungus with self-fertile spores' to PLOS Genetics.**

**The manuscript was fully evaluated at the editorial level and by three independent peer reviewers including the original reviewers. The reviewers appreciated the attention to an important topic but identified some minor concerns that we ask you address in a revised manuscript.

We therefore ask you to modify the manuscript according to the review recommendations. Your revisions should address the specific points made by each reviewer.

In addition we ask that you:
1) Provide a detailed list of your responses to the review comments and a description of the changes you have made in the manuscript.
2) Upload a Striking Image with a corresponding caption to accompany your manuscript if one is available (either a new image or an existing one from within your manuscript). If this image is judged to be suitable, it may be featured on our website. Images should ideally be high resolution, eye-catching, single panel square images. For examples, please browse our** [**archive**](https://journals.plos.org/plosgenetics/volume)**. If your image is from someone other than yourself, please ensure that the artist has read and agreed to the terms and conditions of the Creative Commons Attribution License. Note: we cannot publish copyrighted images.**

**We hope to receive your revised manuscript within the next 30 days. If you anticipate any delay in its return, we would ask you to let us know the expected resubmission date by email to plosgenetics@plos.org.

If present, accompanying reviewer attachments should be included with this email; please notify the journal office if any appear to be missing. They will also be available for download from the link below. You can use this link to log into the system when you are ready to submit a revised version, having first consulted our [Submission Checklist](http://journals.plos.org/plosgenetics/s/submit-now#loc-submission-checklist).

While revising your submission, please upload your figure files to the** [**Preflight Analysis and Conversion Engine**](http://pace.apexcovantage.com/) **(PACE) digital diagnostic tool. PACE helps ensure that figures meet PLOS requirements. To use PACE, you must first register as a user. Then, login and navigate to the UPLOAD tab, where you will find detailed instructions on how to use the tool. If you encounter any issues or have any questions when using PACE, please email us at figures@plos.org.

Please be aware that our** [**data availability policy**](http://journals.plos.org/plosgenetics/s/data-availability) **requires that all numerical data underlying graphs or summary statistics are included with the submission, and you will need to provide this upon resubmission if not already present. In addition, we do not permit the inclusion of phrases such as "data not shown" or "unpublished results" in manuscripts. All points should be backed up by data provided with the submission.**

**To enhance the reproducibility of your results, we recommend that you deposit your laboratory protocols in protocols.io, where a protocol can be assigned its own identifier (DOI) such that it can be cited independently in the future. Additionally, PLOS ONE offers an option to publish peer-reviewed clinical study protocols. Read more information on sharing protocols at** [**https://plos.org/protocols?utm_medium=editorial-email&utm_source=authorletters&utm_campaign=protocols**](https://plos.org/protocols?utm_medium=editorial-email&utm_source=authorletters&utm_campaign=protocols)

**Please review your reference list to ensure that it is complete and correct. If you have cited papers that have been retracted, please include the rationale for doing so in the manuscript text, or remove these references and replace them with relevant current references. Any changes to the reference list should be mentioned in the rebuttal letter that accompanies your revised manuscript. If you need to cite a retracted article, indicate the article’s retracted status in the References list and also include a citation and full reference for the retraction notice.**

**PLOS has incorporated** [**Similarity Check**](http://www.crossref.org/crosscheck.html)**, powered by iThenticate, into its journal-wide submission system in order to screen submitted content for originality before publication. Each PLOS journal undertakes screening on a proportion of submitted articles. You will be contacted if needed following the screening process.

To resubmit, you will need to go to the link below and 'Revise Submission' in the 'Submissions Needing Revision' folder.


Please let us know if you have any questions while making these revisions.  At this stage, no further review will be needed, but we wanted you to have the opportunity to address the final points raised by the reviewers through revision.

Yours sincerely,

Joseph Heitman, MD, PhD
Academic Editor
PLOS Genetics

Kirsten Bomblies
Section Editor
PLOS Genetics


Reviewer's Responses to Questions**

**Comments to the Authors:
Please note here if the review is uploaded as an attachment.**

**Reviewer #1: The authors have addressed my concerns, and the revised manuscript is much clearer now.
One point for which I would like to see some more detail is the analysis of the gene disruptions etc. that the authors detected at the edges of the non-recombining regions. This is quite an interesting point since it strengthens the argument for the hypothesis proposed in Jay et al. (2022), but Figure 5A only shows a broad overview. I think that a figure (perhaps in the supplement) showing more details for each of these differences (and their distance from the borders of the evolutionary strata) would be important, also as a point of comparison for future studies.**

>>We have added details the Figure 5B, which we agree is valuable.

**Reviewer #2: The authors have provided a comprehensive explanation for the issues raised during the review process, and including additional analysis in the revised paper, e.g. to show the presence of mutations that would then be sheltered under their hypothesis.

Minor: during proofing, check line 171 to change "like" to "life".**

>> This has been corrected.

**Reviewer #4: The authors have addressed the concerns raised by previous reviewers and the data appear to be solid and interpretations are reasonable. Here are a few suggestions to improve the manuscript.
1. The discussion is too long and often redundant with the corresponding result sections. This makes the manuscript really long for a read.**

>> We have shortened the discussion by deleting some redundant information. Some points explained in the introduction are a bit re-explained in the discussion, but these are points that are difficult to understand for readers so that we think it is useful to clarify them again before discussing these phenomena.

**2. The authors could also include discussions on recombination suppression observed in bipolar basidiomycetes where dikaryotic stage is long. The authors mentioned "Basidiomycete fungi are dikaryotic across most of their like cycle, but they often
have multi-allelic mating-type loci (50), allowing the loss of those with lower fitness."**

>> We have rephrased the sentence L180-184 to clarify that this explains the case of bipolar basidiomycetes.

**3. Please check the manuscript for some spelling errors and missing periods.**

>> We have carefully checked the manuscript for spelling and punctuation.

**4. Maybe the authors could stress that the recombination suppression is only observed in this particular heterokaryotic strain and also based on its progeny from selfing. Ideally, the phenomenon should be confirmed with additional genetically different heterokaryptic strains.**

>>We have added a sentence in the first paragraph of the discussion.
